# Subacute transverse myelitis with optic symptoms in neuroborreliosis: a case report

**DOI:** 10.1186/s12883-020-01816-y

**Published:** 2020-06-13

**Authors:** Mikolaj Opielka, Witold Opielka, Bartosz Kamil Sobocki, Anna Starzynska

**Affiliations:** 1 Medical University of Gdansk, Gdansk, Poland; 2 Department of Neurology of Hospital No. 1 in Bytom, Bytom, Poland; 3 Department of Oral Surgery, Medical University of Gdansk, Gdansk, Poland

**Keywords:** Lyme disease, *B. burgdorferi* infection, Subacute transverse myelitis, Optic papilla oedema

## Abstract

**Background:**

Subacute transverse myelitis is one of the late manifestations of neuroborreliosis with only a few cases described to the present day.

**Case presentation:**

We present magnetic resonance imaging, cerebrospinal fluid, and electroneurography findings of a young female patient suffering from neuroborreliosis-associated transverse myelitis with a wide constellation of symptoms including papilloedema. Magnetic resonance imaging of the cervical spine has shown an enlargement of the spinal cord in the mid-cervical region. Cerebrospinal fluid findings included lymphocytic pleocytosis, increased levels of anti - Borrelia antibodies, and increased intrathecal anti -Borrelia antibody index. Following the 28-day course of intravenous ceftriaxone, the patient attained complete recovery.

**Conclusions:**

Subacute transverse myelitis in the course of neuroborreliosis should be considered in the differential diagnosis of patients with abnormal magnetic resonance scans of the spinal cord, lymphocytic pleocytosis, and intrathecal antibody production, especially in the tick-endemic areas, even if the tick bite was not reported. Infrequent accompanying symptoms such as papilloedema are diagnostically challenging and cannot be treated as clinching evidence.

## Background

The main established cause of Lyme disease in North America is a spirochete *Borrelia burgdorferi*, whereas in Europe at least five species (*B. afzelii*, *B. garinii*, *B. burgdorferi*, *B. spielmanii* and *B. bavariensis*) were found to cause the disease [1]. The most common early clinical manifestation of Lyme disease is erythema migrans [2]. Other clinical manifestations are arthritis, myocarditis, cranial neuropathy, lymphocytic meningitis, and/or multifocal inflammation of the nervous system including subacute or acute myelitis. Subacute transverse myelitis (SaTM) can reveal in multiple ways involving pyramidal, sensory and autonomic dysfunction of different degrees. Because of the low sensitivity of culture and polymerase chain reaction (PCR), the preferred diagnostic factor is the measurement of IgM and IgG antibody production against *Borrelia* in serum and cerebrospinal fluid (CSF) by enzyme-linked immunosorbent assay (ELISA). Western blot test is performed to confirm positive ELISA results [3]. The major diagnostic tools for transverse myelitis are contrast-enhanced magnetic resonance imaging (MRI) of the spinal cord and signs of inflammation within the CSF [4]. Antibiotic treatment is strongly recommended for Lyme neuroborreliosis (LNB). The most advisable is a 14-day penicillin or ceftriaxone intravenous administration. Orally administrated doxycycline provides equal efficacy [5]. Literature encompassing Lyme disease is well-developed, but case reports with such symptoms as acute transverse myelitis or SaTM in LNB are extraordinarily rare [6, 7].

## Case presentation

A 23-year-old Caucasian female patient was admitted to the Department of Neurology at the end of September due to hands tremor and paresthesia extending to forearms, without the complaint of upper limb weakness. Another major symptom was severe pain in the mid-cervical region. Moreover, the patient suffered from episodes of nausea, vertigo in the period from May to September. During that period the patient also experienced transient episodes of diplopia on distance fixation. Most of the listed symptoms disappeared or decreased their intensity in September except for the limb tremor, episodes of pain in the cervical region, and diplopia.

The medical interview revealed a 2-day episode of fever in May. At that time, the patient could have been exposed to a tick bite in the forest endemic region. However, the tick bite was not remembered. The patient family history was negative for neurological or other chronic familial diseases. She was not taking any medications permanently and did not smoke cigarettes nor consume alcohol or drugs. There was no history of trauma, infections, intoxication and the patient was otherwise in good health. Apart from that, the review of the patient’s systems was negative.

On neurological examination, the muscle strength in the upper right limb was slightly reduced (grade 4 in Lovett scale) in comparison to the left limb. The muscle tone of the lower and upper extremities was at a normal range. Symmetrical intention tremor was observed in her hands, extending periodically to forearms and arms. Normal deep tendon reflexes occurred symmetrically in both upper and lower limbs. The patient’s movements were coherent. The sensory examination did not reveal skin hyperaesthesia in the upper nor lower extremities or spinal tenderness. The sensation was normal in the upper and lower extremities. There were no signs of cranial nerve impairment. The patient was conscious without signs of any psychological or mood disorders**.**

**Fig. 1:**
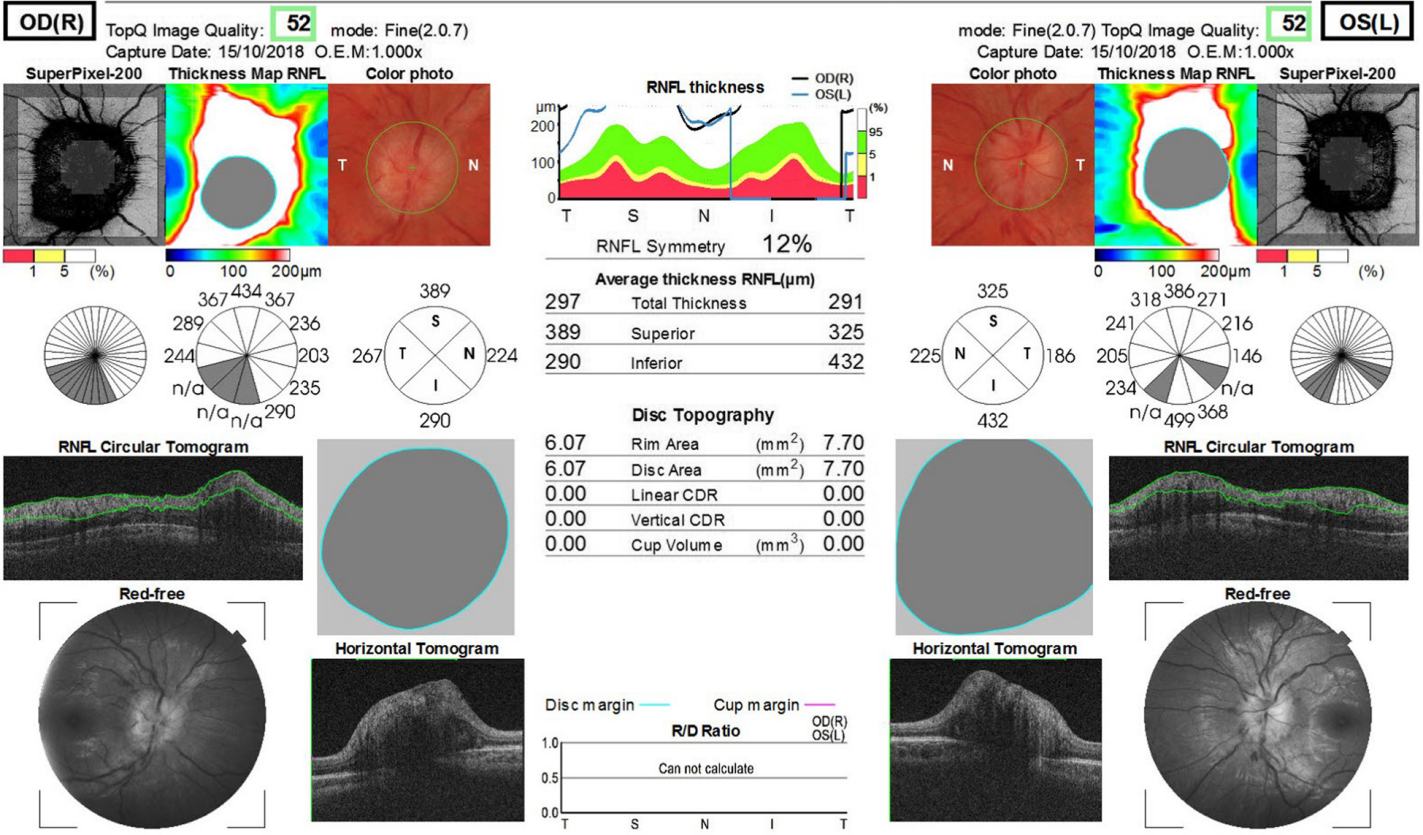
Bilateral, diffuse thickening of RNFL in all quadrants

The patient underwent a comprehensive ophthalmological examination during hospitalization. Her best-corrected visual acuity was measured at 20/20 in both eyes. Pupils were equal, round, and reactive to light. Intraocular pressure was 18 mmHg in both eyes. No aberrations were observed in the anterior segments of the eyes. Besides the fact that the patient complained of transient episodes of diplopia on distance fixation, during the examination the extraocular muscle movements were normal. The fundoscopic examination showed bilateral papilloedema. Blurred optic margins and several flame-like peripapillary hemorrhages were observed in both eyes. The foveal reflex was normal. Optical coherence tomography (OCT) testing showed bilateral diffuse thickening of the retinal fiber nerve layer (RNFL) in all quadrants. The average RFNL was 297 μm in the right eye and 291 μm in the left eye (Fig. 1). The retinal architecture was normal. Automated perimetry visual field test demonstrated no defects.

Nerve conduction study (NCS) was performed for a better evaluation of the cause of the limb numbness and tremor. A Median nerve motor NCS revealed an abnormal reduction of compound muscle action potential (CMAP) in the right median nerve compared to the left median nerve. Reduced amplitude of CMAP was also detected in both peroneal nerves. The reduction of CMAP was more severe in the right peroneal nerve. After supramaximal stimulation F - waves were recorded from median nerves. Decreased F - wave persistence(45%) was observed in the right median nerve. In both peroneal nerves, the absence of F - waves were found. During the orthodromic sensory study, a decreased conduction velocity was observed in the right median nerve and both sural nerves. Based on evidence from an NCS, radiculopathy of nerve roots of both peroneal nerves and the right median nerve was diagnosed. Furthermore, sensory neuropathy of both sural nerves and the right median nerve was also detected.

Routine blood tests were in the normal range. CRP, ESR, and TSH were within physiological limits. VDRL, HIV, EBV, HCV/HBV, HSV, CMV were negative. The patient was AQP − 4 - IgG negative. The ANA test was negative. Vitamin B12 level was in the normal range. High titers of Bd IgM and IgG antibodies were detected in serum using ELISA method. The results were confirmed by Anti Borellial IgG and IgM antibodies detection by western blot method (Table 1).

**Table 1:** Summary of the patient’s CSF and serum parameters. Samples collected on the day of the acceptance and during follow-up visits

Date of samples collection	Admission	After Antibiotic Treatment	1 - Month Follow - up	6 - Month Follow - up	Reference Values
**CSF**					
**CSF cell count/mm**^**3**^**(%lymphocytes)**	323 (70%)	15 (70%)	5 (70%)	4 (70%)	
**Protein level [g/L]**	2.15	0.68	0.58	0.43	0,15 - 0,45
**Albumin [mg/l]**	1374	408	299	–	0.0–350.0
***Bb*IgM [AU/mL]**^**a**^	105.7	75,750	63.4	34.068	< 2.5
***Bb*IgG [AU/mL]**^**a**^	238.8	240	74.43	26.64	< 10
***Bb*- specific AI**	> 1.5	> 1.5	> 1.5	> 1.5	0.0–1.3 - negative > 1.5 - positive
**Serum**					
**Serum IgG against VIsE/C6 peptide [RU/ml]**^**b**^	1462	472	–	130.99	< 20
***Bb*IgG [AU/mL]**^**a**^	154.8	200.7	71.3	53.00	< 4.5
***Bb*IgM [AU/mL]**^**a**^	98.62	87.96	80.31	52.32	< 18
**Negative diagnostic tests performed**					
**Serum AQP-4 IgG**	- VDRL			- HCV Ab	
HIV Ag/Ab	- HSV 1/2 IgM			- HBV Ab	
CMV IgM	- EBV IgM			- ANA	

^a^ Tests performed using ELISA method (Euroimmun,Wroclaw, Poland, Cat. EI 2132–9601-2 G)

^b^ Approximately 4-fold or higher decrease in VIsE IgG titre during 6 month period after antibiotic treatment indicating the effectiveness of treatment (Euroimmun,Wroclaw, Poland, Cat. EI 2132–9601-2 G). *Ab* antibody, *Ag* antigen, *AI* Intrathecal Specific Anitbody Index, *Bb Borellia burgderoferi*, *CMV* Cytomegalovirus, *HSV* Herpes Simplex Virus, *HBV/HVC* Hepatitis B/C virus, *EBV* Epstein - Barr Virus, *HIV* Human Immunodeficency Virus, *VDRL* Veneral Research Disease Laboratory Test, *AQP - 4* Aquaporin 4, *ANA* Antinuclear Antibodies

**Fig. 2:**
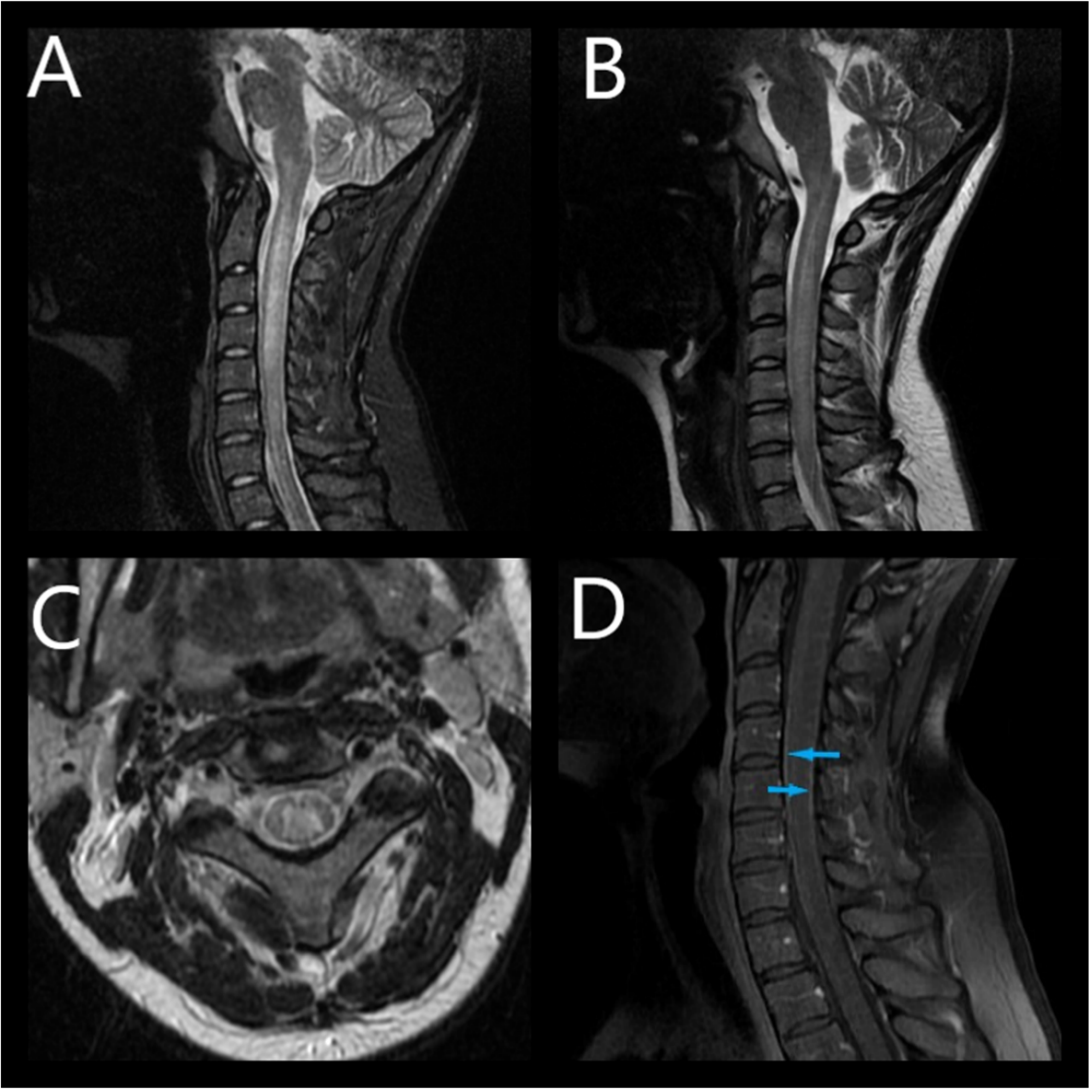
Longitudinally extensive enlargement of the spinal cord with a hyperintense lesion in the central part of the spinal cord on T2(**b**, **c**) and STIR – weighted (**a**) images. Post-contrast meningeal enhancement in T1 (**d**)

**Fig. 3:**
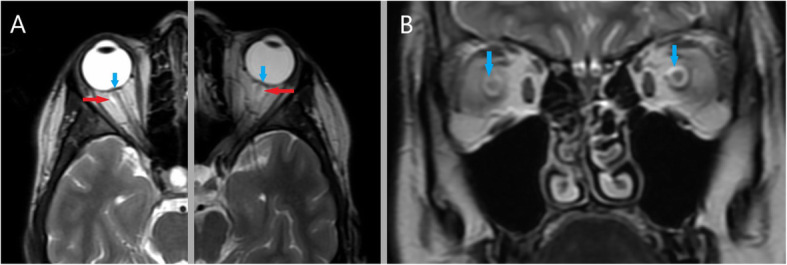
Bilateral optic disc protrusion and hyperintense perioptic nerve sheath in T2- weighted fat-saturated image

MRI of the cervical spine was obtained to determine the nature of the patient’s tremor and pain in the cervical region. MRI images showed longitudinally extensive (> 3 segments) enlargement of the spinal cord mostly visible from C3 to C6/C7 level. T2 and STIR-weighted images demonstrated a hyperintense, spindle-like lesion in the central part of the spinal cord extending from C1 to C6/C7 without enhancement in the post-contrast image. CSF reservoirs were constructed. Contrast-enhanced T1 images revealed signal amplification in the meninges (Fig. 2). The MRI of the optic nerve disclosed bilateral protrusion of the optic nerve heads, slight vertical tortuosity of both optic nerves, and bilateral hyperintense perioptic nerve sheath in T2 images (Fig. 3). Brain MRI showed an empty sella turcica sign. Together these signs could indicate elevated intracranial pressure.

After the analysis of MRI images, the patient had a lumbar puncture performed. CSF findings included lymphocytic pleocytosis, increased level of protein, and albumin. Anti - Bb IgM and IgG antibodies were detected in CSF using Western Blotting. The titers of anti -Bb IgM and IgG antibodies were significantly increased. Bb - specific antibody (AI) index was elevated indicating the intrathecal production of antibodies (Table 1).

**Fig. 4:**
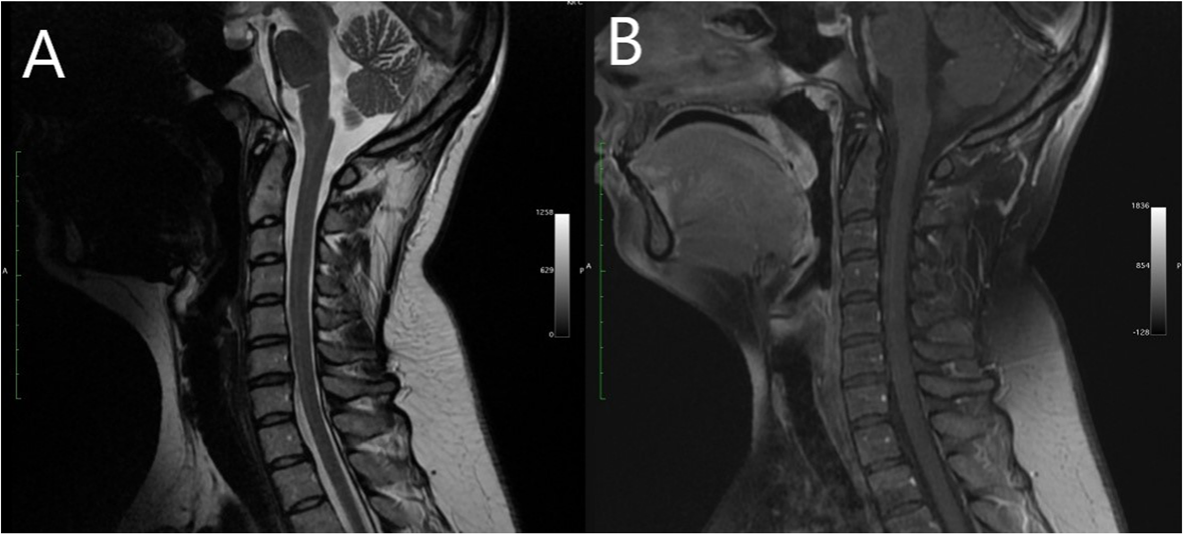
Complete resolution of the lesion (**a**) and lack of meningeal enhancement after antibiotic treatment (**b**)

**Fig. 5:**
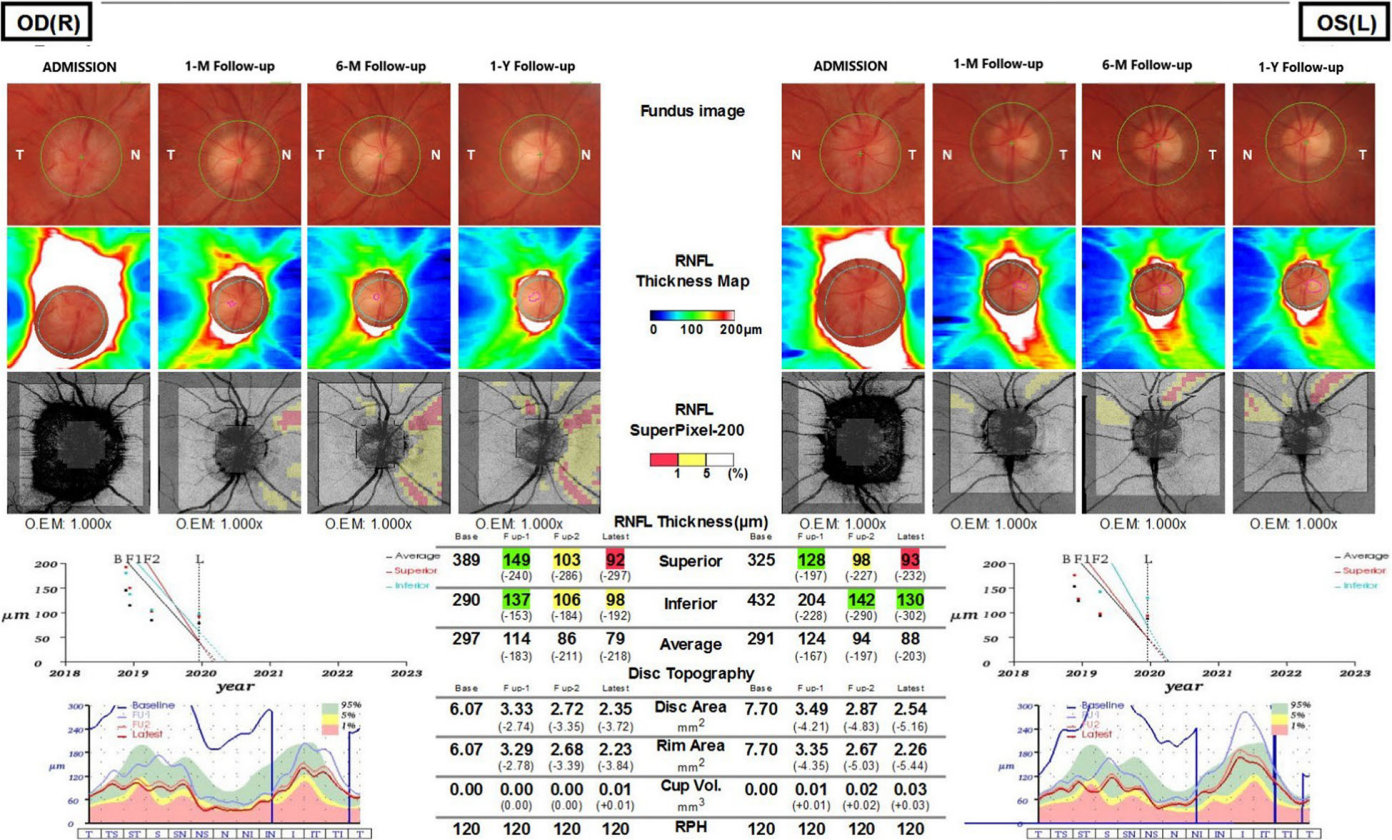
A gradual decrease of RNFL swelling observed in 1 – year follow – up, leading to atrophy of RNFL, predominantly I nasal quadrant of the right eye

The patient met the EFNS criteria for definite LNB [8] and the diagnosis was SaTM due to Bb infection. She received a 28-day course of ceftriaxone (2 g/day intravenously). After the period of antibiotic administration, the symptoms subsided and the patient was discharged from the hospital. The patient’s CSF findings, including pleocytosis, the levels of albumin and protein normalized. Bilateral swelling of optic discs decreased. Post-treatment MRI images showed no abnormalities in the spinal cord and meninges (Fig. 4). All other symptoms had subsided except for minor tremors in the left hand, which vanished one month later. The patient was monitored during regular follow - up visits for one year. Six months after the antibiotic treatment the patient’s anti C6 antibody titer decreased by 11 - fold indicating the effectiveness of antibiotic treatment [9] (Table 1). During follow -up visits no additional aberrations were found in MRI scans. In one year follow up a gradual decrease in swelling of RLNS was observed leading to optic atrophy in superior and nasal quadrants of the right eye and in the superior quadrant of the left eye (Fig. 5).

## Discussion and conclusion

Transverse myelitis can be described as a clinical syndrome, of various aetiology, causing bilateral spinal injury due to an inflammatory response of different duration. The term “subacute transverse myelitis” is defined as an inflammatory subtype of transverse myelitis with longer onset (weeks) in comparison with acute transverse myelitis (a couple of hours to several days) [10]. The typical manifestations of transverse myelitis include bilateral (frequently unsymmetrical) or unilateral limb weakness, sensory disturbances, and disruption of the autonomic system below the affected level of the spinal cord [11]. The pathophysiological characteristics of transverse myelitis include focal concentrations of lymphocytes with monocytes, astroglial, and microglial activation inside the spinal cord, and demyelination of different degrees leading to axonal degeneration [12, 13]. Moreover, processes like molecular mimicry causing the production of autoantibodies can also lead to the development of transverse myelitis. Therefore, the diagnostic hallmarks of TM are intrathecal production of antibodies or pleocytosis and spinal cord inflammation revealed on gadolinium-enhanced MRI images [14]. It is estimated that the occurrence of TM ranges from 1.3 to 8 cases per million, affecting predominantly individuals in two age groups – between 10 to 19 years of age and between 30 to 39 years [12]. TM typically stems from ischemia, infections, paraneoplastic processes, and autoimmunological diseases including Sjögren’s syndrome, systemic lupus erythematosus (SLE), sarcoidosis, Behçet’s disease, or antiphospholipid syndrome (APS). TM can be also one of the initial symptoms of multiple sclerosis, neuromyelitis optica (NMO), or neuromyelitis optica spectrum disorders (NMOSD) [15, 16].

It is estimated that transverse myelitis with infectious or parainfectious aetiology accounts for 12% of transverse myelitis cases [17]. The most significant identified agents include bacteria e.g. *Mycoplasma pneumonia*, *B. burgdorferi*, *Treponema pallidum*, *Mycobacterium tuberculosis*; viruses, e.g. EBV, HSV-1, HSV-2, VZV, CMV, HIV; fungi, e.g. *Blastomyces*, *Coccidioides*, *Aspergillus*, *Candida*; and parasites, e.g. *Toxoplasma gondii* [13].

Our patient presented typical manifestations of SaTM with segmental swelling and enlargement of the spinal cord. The patient met the EFNS criteria for definite neuroborreliosis, thus we could classify the patient’s case as a disease-associated SaTM with borrelial aetiology [8].

Neuroborreliosis is defined as an infection of the nervous system caused by *B. burgdorferi, B. afzelii, B. garinii* or *B. bavariensis*, *w*hich can be classified as a disseminated stage of Lyme disease and can be later subdivided into early(< 6 months) and late(> 6 months) phases [16]. The neurological manifestations of disseminated Lyme disease are observed in 10–15% of all patients with Lyme disease in both Europe and the USA [2]. LNB may be the first and only clinical sign in patients with early Lyme disease. Predominantly early LNB develops within 2–3 weeks after the infection. The precise time of the infection is frequently unknown because only about one-third of the patients can recall a tick bite, whereas only half of them remember the appearance of skin lesions similar to erythema migrans [17]. The major clinical manifestations of early LNB include peripheral radiculitis, cranial neuritis – predominantly facial palsy, and lymphocytic meningitis, which is the most common single cause of LNB. In the adult European population LNB most commonly presents as painful meningoradiculitis (Bannwarth’s syndrome), which causes severe, migrating radicular pain, accompanied by peripheral nerve paresis, uni- or bilateral facial palsy and CSF pleocytosis [2]. Less frequent PNS manifestations include plexitis and mononeuritis multiplex. CNS involvement is rarely observed and may manifest as encephalitis or acute/subacute TM. Another rare and frequently overlooked aspect of LNB is optic nerve involvement [18]. Typical opthalmological symptoms include blurred vision, strabismus, diplopia, and variable affection of abducens nerve. Papilloedema in the course of LNB was observed mainly in children, while adult occurrence is uncommon. It can be associated with raised intracranial pressure (ICP) or different forms of optic neuropathy including papillitis or retrobulbar neuritis [19].

The clinical presentation of our patient was diagnostically challenging. The only indicator of a possible tick bite was an episode of raised temperature, followed by symptoms of neck stiffness and pain reported by the patient. Another diagnostic challenge included a relatively long time interval between first symptoms appearance and hospitalization (5/6 months) during which the patient could develop the chronic phase of LNB. After analysing the spinal cord lesion, compressive myelitis was excluded. Due to multisegmental (> 3 vertebral body lengths), diffuse character of the lesion it met the criteria of longitudinally extensive transverse myelitis (LETM), thus the patient was tested for some typical viral, bacterial and autoimmunological factors that cause LETM [20]. Notably, NMO and neuromyelitis optica spectrum disorders (NMOSD) were included in the differential diagnosis, because of the uncommon combination of optic nerve involvement with LETM. However, the core clinical characteristics for NMOSD including the production of antibodies against AQP − 4, an acute type of transverse myelitis, severe optic neuritis revealed on MRI, area postrema syndrome (APS), brainstem and diencephalic syndromes were absent [21, 22]. Because of the time in which the transverse myelitis developed (weeks), the subacute subtype of transverse myelitis was diagnosed. The patient was a resident of tick - endemic area, thus we performed a comprehensive Lyme disease diagnosis. Pleocytosis, increased level of Bb *-* specific antibodies in CSF, increased AI and negative bacterial and viral tests enabled to diagnose the patient with a definite case of SaTM in the course of LNB [8]. The diagnosis was also strongly supported by the symptoms subsidence after ceftriaxone administration and lack of symptoms relapse in a one-year follow-up. We decided for prolonged 28 - day intravenous ceftriaxone therapy, owing to the long period from infection to hospitalization and unique, extensive combination of symptoms. Furthermore, the specific recommendations for LNB with optic nerve are absent, thus we adopted a similar therapeutic strategy described earlier in the case of the adult patient with isolated LNB - related papilloedema [23].

There was convincing evidence including papilloedema without visual acuity loss, optic nerve MRI changes, and empty sella turcica indicating an occurrence of benign intracranial hypertension in the case of our patient [24]. However, the result was not confirmed by CSF opening pressure. Together with confirmed LNB this evidence strongly suggested a Borrelial infection as a cause of papilloedema.

We present one of the few described cases of SaTM associated with LNB and involving the optic nerve. It is essential to consider SaTM when diagnosing LNB, especially in the endemic regions. Moreover, symptoms associated with optic nerve should also be considered when diagnosing patients with LNB. However, some very strong evidence is required to link papilloedema with LNB, thus it should not be used as the major diagnostic criterion.

## Data Availability

All data and material supporting our findings are contained within the manuscript.

## Abbreviations

LNB: Lyme Neuroborreliosis
SaTM: Subacute Transverse Myelitis
NMO: Neuromyelitis optica spectrum disorders
NMOSD: Neuromyelitis optica spectrum disorders
AQP – 4: Aquaporin 4
LNB: Lyme Neuroborreliosis
Bb: *Borrelia burgderoferi*
AI: Intrathecal specific antibody index
LETM: Longitudinaly extensive transverse myelitis
NCS: Nerve conduction study
CMAP: Compound muscle action potential
OCT: Optical coherence tomography
RFNL: Retinal fiber nerve layer
